# Quantitative perfusion-CMR is significantly influenced by the placement of the arterial input function

**DOI:** 10.1007/s10554-020-02049-3

**Published:** 2020-10-12

**Authors:** Ibnul Mia, Melanie Le, Christophe Arendt, Diana Brand, Sina Bremekamp, Tommaso D’Angelo, Valentina O. Puntmann, Eike Nagel

**Affiliations:** 1 Institute for Experimental and Translational Cardiovascular Imaging, University Hospital Frankfurt, Theodor-Stern Kai 7, 60590 Frankfurt am Main, Germany; 2 Department of Biomedical Sciences and Morphological and Functional Imaging, G. Martino University Hospital Messina, Via Consolare Valeria 1, 98100 Messina, Italy; 3 Institute for Experimental and Translational Cardiovascular Imaging, University Hospital, Goethe University, Frankfurt am Main, Germany

**Keywords:** Cardiovascular magnetic resonance, Myocardial perfusion, Coronary artery disease, Ejection fraction, Heart failure, Signal intensity, Aortic input function

## Abstract

The aim of this study is to provide a systematic assessment of the influence of the position on the arterial input function (AIF) for perfusion quantification. In 39 patients with a wide range of left ventricular function the AIF was determined using a diluted contrast bolus of a cardiac magnetic resonance imaging in three left ventricular levels (basal, mid, apex) as well as aortic sinus (AoS). Time to peak signal intensities, baseline corrected peak signal intensity and upslopes were determined and compared to those obtained in the AoS. The error induced by sampling the AIF in a position different to the AoS was determined by Fermi deconvolution. The time to peak signal intensity was strongly correlated (r^2^ > 0.9) for all positions with a systematic earlier arrival in the basal (− 2153 ± 818 ms), the mid (− 1429 ± 928 ms) and the apical slice (− 450 ± 739 ms) relative to the AoS (all p < 0.001). Peak signal intensity as well as upslopes were strongly correlated (r^2^ > 0.9 for both) for all positions with a systematic overestimation in all positions relative to the AoS (all p < 0.001 and all p < 0.05). Differences between the positions were more pronounced for patients with reduced ejection fraction. The error of averaged MBF quantification was 8%, 13% and 27% for the base, mid and apex. The location of the AIF significantly influences core parameters for perfusion quantification with a systematic and ejection fraction dependent error. Full quantification should be based on obtaining the AIF as close as possible to the myocardium to minimize these errors.

## Introduction

Perfusion cardiovascular magnetic resonance (perfusion-CMR) is an established test for assessing the presence and severity of myocardial ischemia with a class IA indication in the most recent European Guidelines [1]. It has shown to be strongly correlated with fractional flow reserve [2, 3] and has recently been shown to allow for a safe noninvasive guidance of patients with moderate to severe pretest likelihood for coronary artery disease (CAD) which is noninferior to invasive angiography supported by fractional flow reserve [4]. Increasingly fully quantitative methods are employed to determine myocardial blood flow (MBF) and flow reserve [5–8]. Perfusion quantification requires correction for the speed and amount of contrast agent arrival to the coronary arteries with the so called “arterial input function” (AIF). Since the AIF cannot be measured in the coronary arteries with CMR, the use of the same imaging plane as the perfusion measurement itself (e.g. basal short axis view, mid short axis view or apical short axis view) or the use of the basal short axis plane for all other slices has become the accepted standard [9]. As the contrast agent bolus disperses during its passage through the circulation some influence of the location of the AIF on the final results is to be expected.

However, the influence of the location on the AIF has not been systematically assessed. We determined the presence and extent of error induced by positioning the AIF in the basal, mid and apical left ventricle, rather than the aortic sinus (AoS) which is closest to the coronary ostiae. For quantification we used Fermi deconvolution. It is currently the most widely used convolution method.

## Methods

In 39 patients with suspected nonischemic cardiomyopathy and a wide range of ejection fractions a weight adapted contrast agent bolus (0.00375 mmol/kg body weight in 95% saline, Gadovist R, Bayer AG, Leverkusen, Germany) was injected with a flow rate of 3 ml/sec followed by a 20 ml saline chaser (Accutron MR, Medtron, Saarbrücken, Germany) (adapted from [10]). During the first pass of the contrast agent a 3-chamber view was obtained in mid-diastole of every heartbeat using the identical sequence usually used for stress-perfusion imaging in our Institute (Echo-Time: 1.11-2.0 ms, Repetition-Time: 3.5 ms, Flip Angle: 35–50°, typical acquired spatial resolution 2. × 2.5 × 8 mm, 3 T, 90° saturation prepulse, with 100 ms prepulse delay ) with the patients holding their breath in expiration. Two proton density images per slice were acquired at the beginning of each acquisition for estimating the coil sensitivity profile [11].

All patients were scanned at 3 T (Skyra, Siemens AG, Erlangen, Germany) using 32-channel surface coils. All patients gave written informed consent and the study was approved by the local ethics committee (T1 Mapping Registry CMR Studie, Business Nr.: 1/16 and Decipher HFpEF, Business Nr.: 273/17).

### Image analysis

**Fig. 1:**
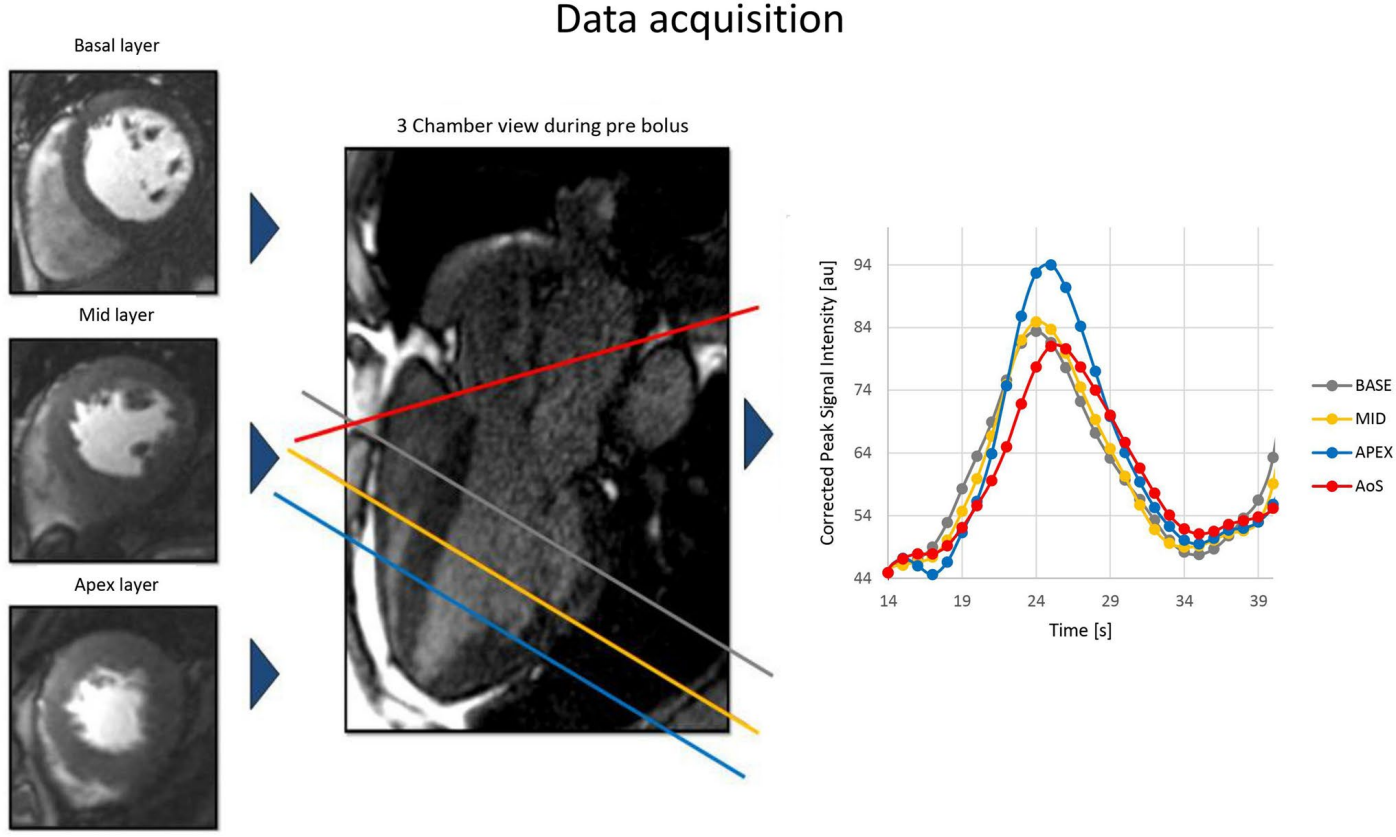
Data acquisition: the three typical regions of interest (basal, mid, apex, defined as 25%, 50% and 75% of the end-systolic distance from the mid of the mitral valve to the tip of the apical blood pool) plus a fourth ROI (aortic sinus) were placed in each dynamic of the first pass perfusion 3-chamber image. After baseline correction, signal intensity time curves were calculated, resulting in four aortic input functions

Using MEDIS Suite 3.2 (Medis, Leiden, The Netherlands), three regions of interest (ROI) were placed in each dynamic of the first pass perfusion 3-chamber images at a basal, mid and apical level. These levels are defined as 25%, 50% and 75% of the endsystolic distance from the mid of the mitral valve to the tip of the apical blood pool of the LV blood [12]. These positions are routinely used for the position of the short axis views for perfusion imaging in our Institute. Care was taken to avoid any papillary muscles. A fourth ROI was placed in the aortic sinus (AoS) at the level of the coronary arteries (Fig. 1). After baseline correction, signal intensity time curves were constructed for each ROI generating four AIFs. Time to peak was determined as the time between arrival of the contrast agent (signal increase above 2 SD of the contrast free images) and the time of the peak signal intensity. The change of signal from baseline to peak signal intensity was determined and noted as peak signal intensity. Upslope was defined as the highest upslope of three consecutive heartbeats of the AIF. For quantitative analysis Fermi deconvolution was performed with R studio 1.2.5001 using a least square fit based on the Levenberg-Marquardt algorithm.

Differences versus the AIF in the AoS were then calculated in [ms] for time to peak and in [%] for peak signal intensity and the upslope.

To calculate the error induced by the different locations, four average AIFs of all patients were calculated after baseline correction and normalization for time to peak and peak signal intensity using the data obtained from the four sampling positions. A single myocardial response function was created from a patient with an ejection fraction closest to the mean of all patients. The difference in MBF based on the AIF obtained in the three ventricular positions versus the AIF obtained in the AoS was calculated and provided in [%].

Ejection fraction was measured using automatically generated contours with final user correction and approval (SuiteHeart, NeoSoft, Pewaukee, Wi, USA). Patients were categorized into preserved ejection fraction (EF > 50%) or reduced ejection fraction (EF ≤ 50%).

### Statistical analysis

The following statistical tests were used as appropriate: rANCOVA for repeated measurements with F-test for significance, unpaired Welch two sample t-test for differences between group categories, linear regression with correlation coefficient for comparing two continuous variables (Pearson’s method for normally distributed data and Spearman’s method for not normally distributed data as determined by Shapiro-Wilk normality test). All tests were 2-tailed. Statistical significance was reached at p < 0.05. Statistical analysis was performed with R studio (Version 1.2.5001).

## Results

Patient characteristics are shown in Table 1.

**Table 1:** Patient characteristics

Age (years)	62.1 ± 12.1
Female (%)	48.3%
BSA (m/cm^2^)	1.95 ± 0.28
Ejection fraction (%)	46 ± 17

**Fig. 2:**
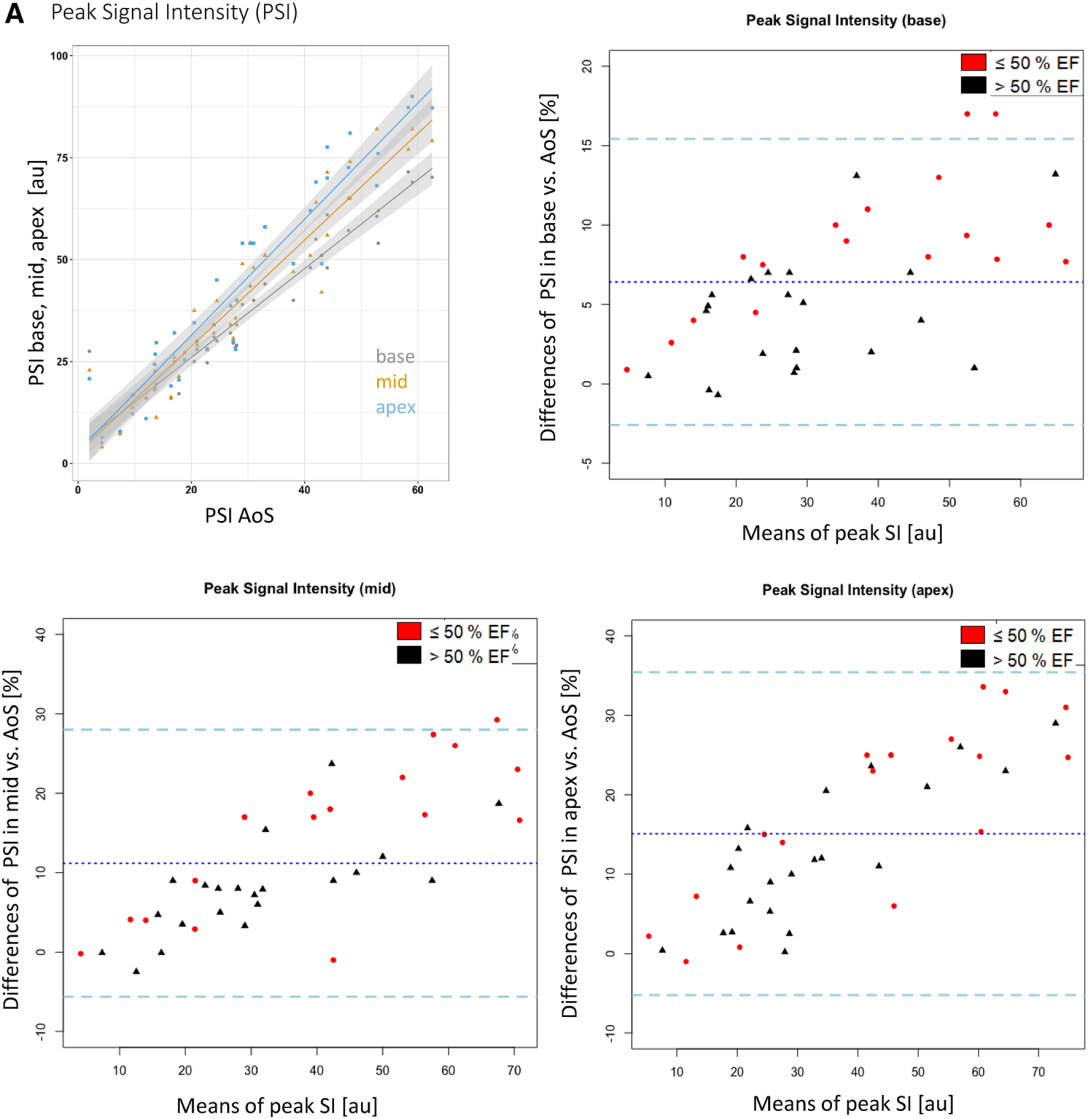

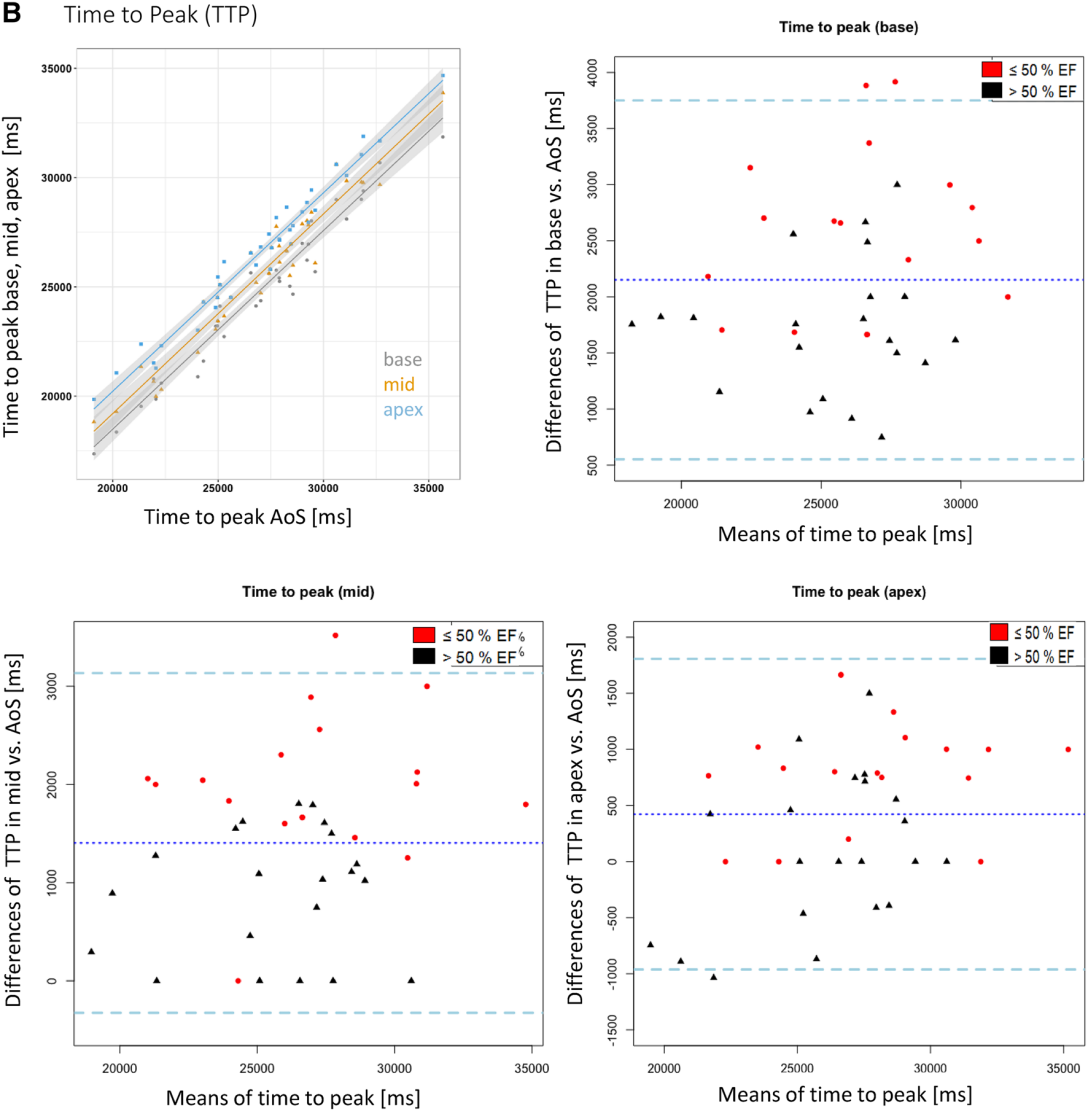

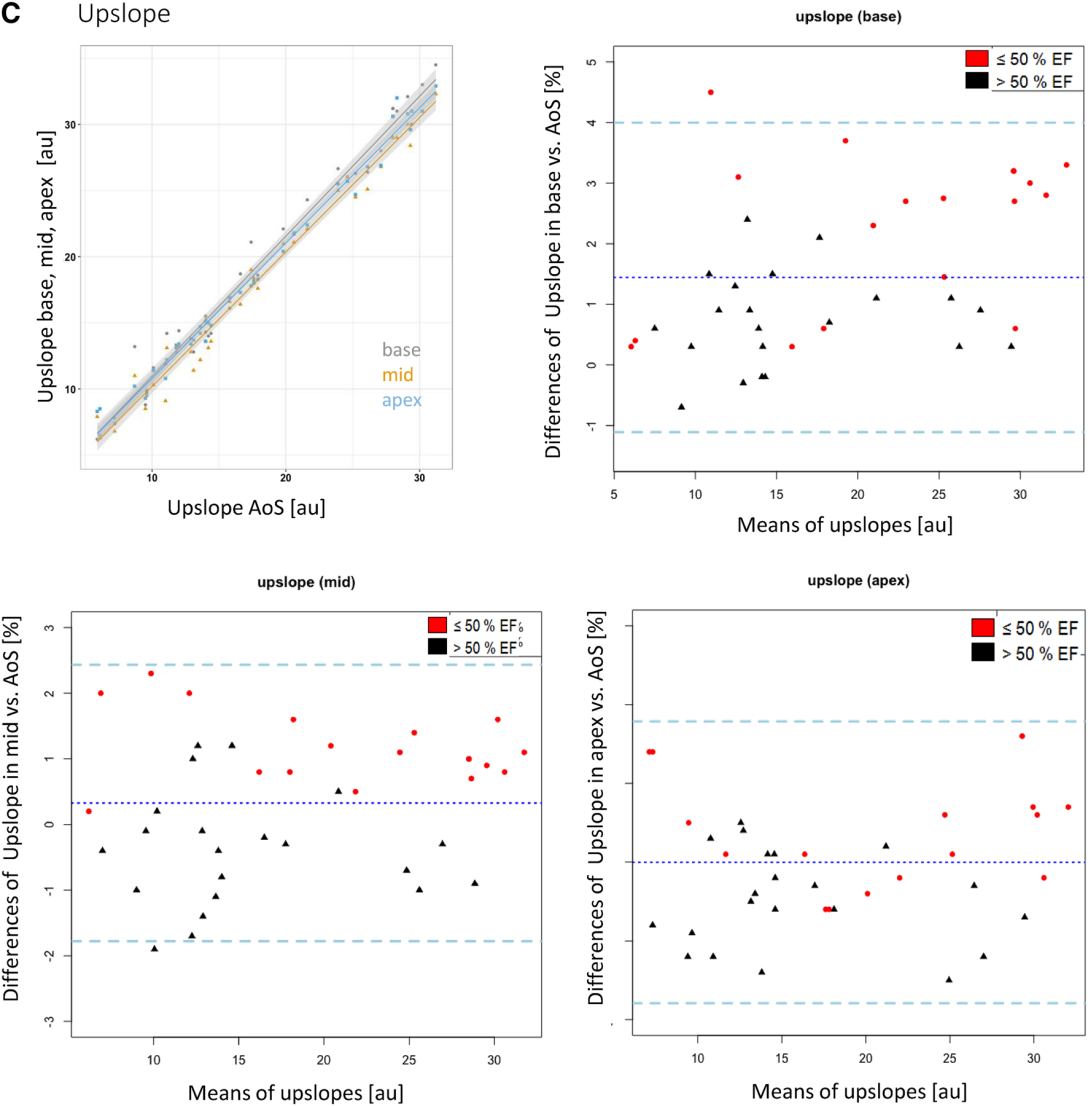
**a** Influence of position and EF on the peak signal intensities: Correlation graph and Bland–Altman plots of the peak signal intensities for each short axis layer (basal, mid, apex). The strong correlation between the data in the AoS and the three other locations (r^2^ > 0.9 for all locations) can be appreciated. The Bland Altman plots show a large bias demonstrating a systematic error. Patients with ejection fraction > 50% are shown with black triangles, those with EF ≤ 50% are shown with red dots. Note the large confidence intervals demonstrating large random errors. The dotted middle line indicates the mean difference of the peak signal intensities. The dashed lines represent the 95% confidence intervals. Values are given in %. **b** Influence of position and EF on time to peak: correlation graph and Bland–Altman plots of the time to peak signal intensities for each short axis layer (basal, mid, apex). Values are given in ms. All other information similar to Fig. 2a. **c** Influence of position and EF on the upslope: correlation graph and Bland–Altman plots of the upslope for each short axis layer (basal, mid, apex). Values are given in %. All other information similar to Fig. 2a

Strong correlations were observed between the respective parameters (peak signal intensity, time to peak, upslope) in the AoS, and the three left ventricular positions (r^2^ between 0.91 and 0.99, p < 0.01 for all, Fig. 2a–c).

Highly significant differences between the AoS and the three other locations were observed for each parameter (all p < 0.001, except for the upslope of the midslice versus AoS with p < 0.05; Table 2).

**Table 2:** Differences relative to the Aortic sinus

	Base	p value	Mid	p value	Apex	p value
Delta time to peak (dTTP) (ms)	− 2153 ± 818	< 0.001	− 1429 ± 928	< 0.001	− 477 ± 741	< 0.001
Delta peak signal intensities (dpSI) (%)	21.8 ± 14.0	< 0.001	40.4 ± 35.3	< 0.001	52.1 ± 27.0	< 0.001
Delta upslope (%)	11.63 ± 16.1	< 0.001	3.0 ± 10.75	< 0.05	6.91 ± 9.17	< 0.001
Delta flow (%)	8		13		27	

The largest differences to the AoS were found for the time to peak at the base, for the peak signal intensity at the apex and for the upslopes at the base (Table 2).

**Fig. 3:**
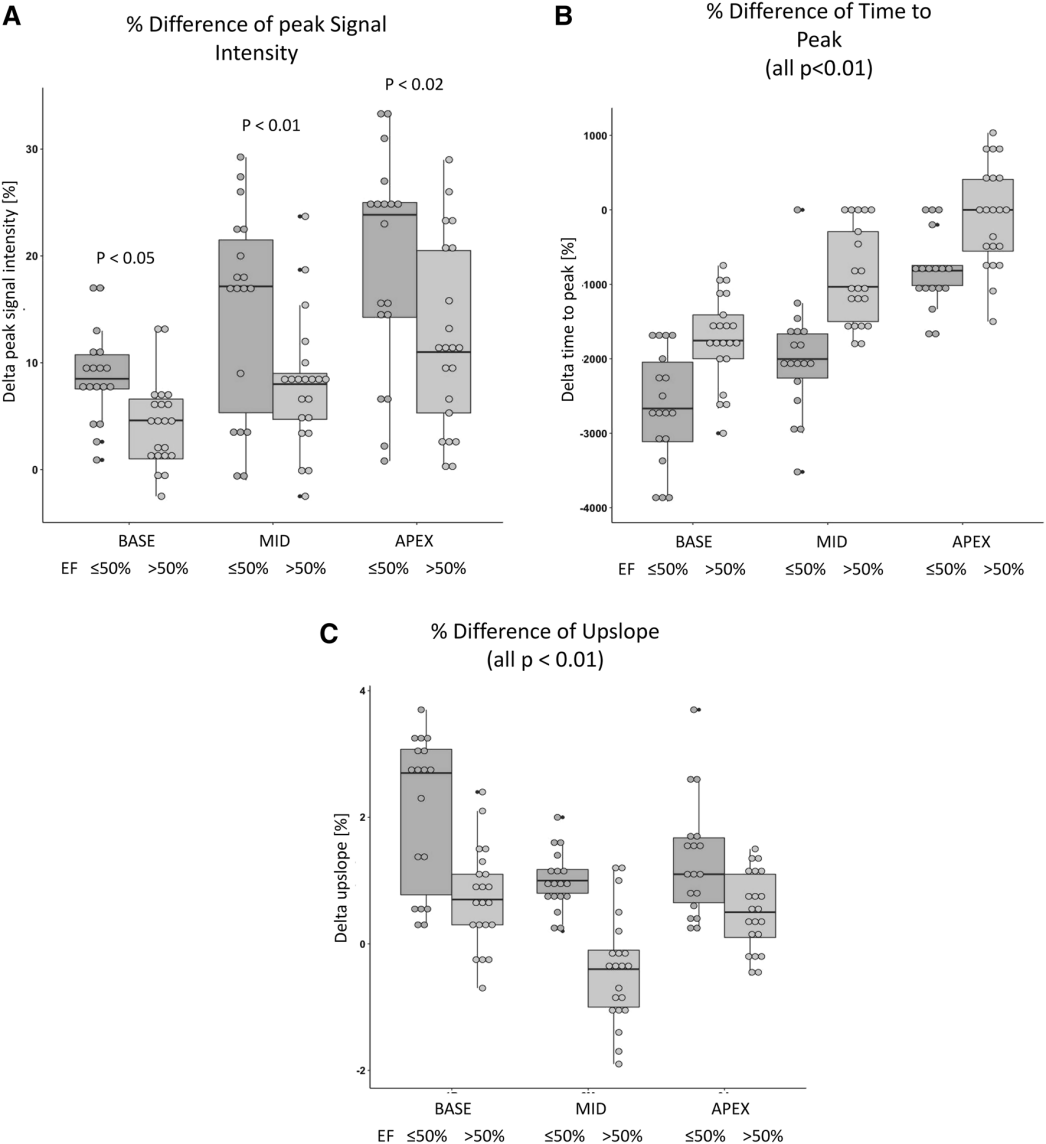
**a** Influence of position and EF on the peak signal intensity: Influence of slice position and ejection fraction (EF) on the peak signal intensities. Patients with and EF > 50% are shown in light grey, patients with an EF < 50% are shown in dark grey. The center line in each box represents the median, the upper and lower border of the box represent the upper and lower quartile respectively and the whiskers represent the values that fall within the 1.5 x interquartile range above and below the respective quartile. **b** Influence of position and EF on the time to peak (all p < 0,01): Influence of the ejection fraction (EF) regarding the time to peak signal intensity in each layer. All other information similar to Fig. 3a. **c **Influence of the position and EF on the upslope: Influence of the ejection fraction (EF) regarding the upslope in each layer. All other information similar to Fig. 3a

All differences were significantly more pronounced in patients with reduced LVEF in comparison to patients with preserved LVEF (Fig. 3a–c).

The differences for the shape of the input function resulted in errors of 8%, 13% and 27% for the base, the mid slice and the apex in comparison to the AoS.

## Discussion

Our data shows, that the position of the region of interest used to determine the arterial input function (AIF) for perfusion quantification has significant effects on its shape. Some of these differences are systematic, however, there is also large random error. Most importantly, the magnitude of the error correlates negatively with the ejection fraction resulting in larger errors in patients with reduced ejection fraction. This is of specific importance, as one of the core reasons to use the AIF is to control for differences of contrast agent delivery in patients with reduced ejection fraction.

For two decades myocardial perfusion-CMR studies were mainly reported visually by assessing the speed and amount of signal increase within the myocardium during the first arrival of the contrast agent bolus during maximal vasodilation. Until recently, most attempts to quantify MBF based on perfusion-CMR were restricted to academic research sites and the effort for full quantification, in combination with a large amount of assumptions, was tedious. If quantification was performed, frequently a semiquantitative approach based on differences of specific parameters (e.g. upslope) between stress and rest was used. This has the advantage to cancel out some of the systematic effects. In addition, local normal ranges or a comparison between segments of a given patient were applied. These approaches, however, have the disadvantage to not allow provision of absolute ranges of normal with objective and transferable cut-off values, require stress and rest imaging and are limited in their ability to address changes, e.g. with therapy. More recently fully quantitative methods have become readily available and started to be used in clinical practice. However, when providing full quantification of MBF in ml/g/min myocardium any error will be misleading, as the claim for an absolute number inherently infers, that all assumptions in the processing chain of the parameter are sufficiently controlled.

The role of the position of the input function has been analyzed previously, mainly for cardiac positron emission tomography where a similar dependency of MBF on the position of the input function was found [13, 14]. Its importance for perfusion CMR has been discussed [15], but not systematically analysed.

The obtained data in the current report provide important knowledge:

First, the position of the AIF creates a systematic error. This error could be addressed by either using normal values for the specific pathway used to obtain the results, by applying correction factors or potentially, by calculating a myocardial perfusion reserve based on the quantitative data, rather than providing MBF itself. The excellent correlation between the various locations for each parameter demonstrate, that the systematic effect is strong and can be accounted for. Importantly, the largest systematic difference was found for time to peak, which is least important for full quantification.

Second, the position of the AIF creates a random error demonstrated by the large confidence intervals in the Bland–Altman analyses. While it may be possible to reduce these effects by better fitting algorithms and modern machine learning optimization, they highlight the need to minimize this error during data acquisition.

Third, the error created by different positions of the AIF varies with important core parameters, such as ejection fraction. Again, it may be possible to partially account for this by taking the ejection fraction or other parameters of cardiac function into account, this observation is especially disturbing, as one of the core functions of the AIF was to correct for physiological differences, such as cardiac function. Given that normal values for myocardial perfusion are mainly based on healthy controls which also have normal ejection fraction, this error may cause a misunderstanding of underlying pathophysiologies, or systematic misclassification of the presence and extent of myocardial ischemia in specific subgroups of patients.

In order to determine the best AIF, it is necessary to consider and analyze the systematic errors mentioned above and the limitations of the current study mentioned below. Diseases that affect the ejection fraction or cardiac blood flow may have a strong impact on the AIF.

A first opportunity to generate further data in the future is to examine stress images (taking the aspects mentioned above into account) and compare them with the data obtained here. After that a first concept for the standardization of CMR perfusion studies can be proposed.

### Limitations

Several limitations apply. The study was meant to highlight the need to standardize the location of the AIF and obtain it in the aortic sinus whenever possible. As such, we did not address other potential confounders, such as valvular heart disease, arrhythmia, or aortic pathologies. It can be speculated, that even larger errors may be observed in these patient groups. We did not perform stress, calculate diagnostic accuracy, or perform full quantification of MBF. Extending our observations to these topics would add data, but not broaden the core results underlined by strong significant differences. Furthermore, gender-specific differences, e.g. regarding the ejection fraction, were not investigated. Our study did not provide any clear signs for this. Investigating this would be relevant from our point of view, but would go beyond the scope of this scientific work. In addition, due to the small number of patients, an adequate statement to that topic could not be made here.

## Conclusions

The location of the AIF significantly influences core parameters for perfusion quantification such as peak signal intensity, time to peak SI and upslope. The placement of the AIF creates systematic errors, random errors and ejection fraction dependent errors. Full quantification should therefore be based on obtaining the AIF as close as possible to the myocardium to minimize these errors.

## Abbreviations

3Ch: 3-Chamber
AIF: Arterial input function
AoS: Aortic sinus
CAD: Coronary artery disease
CMR: Cardiovascular magnetic resonance
LVEF: Left ventricular ejection fraction
LV: Left ventricle
ROI: Region of interest
MBF: Myocardial blood flow
